# Is Being Employed Always Better for Mental Wellbeing Than Being Unemployed? Exploring the Role of Gender and Welfare State Regimes during the Economic Crisis

**DOI:** 10.3390/ijerph16234799

**Published:** 2019-11-29

**Authors:** Imma Cortès-Franch, Vanessa Puig-Barrachina, Hernán Vargas-Leguás, M. Marta Arcas, Lucía Artazcoz

**Affiliations:** 1 Health and Work Department, Agència de Salut Pública, 08023 Barcelona, Spain; vpuig@aspb.cat (V.P.-B.); hvargas@aspb.cat (H.V.-L.); marcas@aspb.cat (M.M.A.); lartazco@aspb.cat (L.A.); 2 CIBER en Epidemiología y Salud Pública (CIBERESP), 28029 Madrid, Spain; 3 Institute of Biomedical Research (IIB-Sant Pau), 08041 Barcelona, Spain; 4 Department of Pediatrics, Obstetrics and Gynecology, and Preventive Medicine, Universitat Autònoma de Barcelona, 08193 Barcelona, Spain; 5 Center for Research in Occupational Health, Universitat Pompeu Fabra, 08002 Barcelona, Spain

**Keywords:** unemployment, job quality, mental wellbeing, gender, welfare states, Europe

## Abstract

The growth of poor jobs related to economic crisis adds to its increase since the mid-1970s as a result of new forms of flexible employment. In Europe, there is no clear evidence on whether working in a poor-quality job is better for mental wellbeing than being unemployed. The objectives of this study were to compare mental wellbeing between the unemployed and those working in jobs with different quality levels and to examine gender and welfare state differences in Europe. We selected 8324 men and 7496 women from the European Social Survey, 2010. Hierarchical multiple logistic regression models were fitted, separated by sex and country group. No significant differences in mental wellbeing were shown between unemployed-non-active, unemployed-active, and those working in low-quality jobs in either sex. Only men from Conservative countries in low-quality jobs had better mental wellbeing than unemployed (non-active) men. Only having a good-quality job reduced the likelihood of poor mental wellbeing compared with being unemployed (non-active) among men in all countries (except Social-Democratic) and among women in Eastern and Southern European countries. No differences were observed among men or women in Social-Democratic countries, while strong gender differences were found in Conservative and Liberal countries. Our study indicates the need to take job quality into account, in addition to creating jobs during economic crises. The main mechanisms to explain the strong gender and welfare state differences identified could be social protection for unemployed, labor market regulations, and family models.

## 1. Introduction

The global economic crisis has returned the spotlight to the debate on the quantity and quality of jobs, but the introduction of the health perspective in this discussion is fairly recent. Since the beginning of the crisis at the end of 2007, global unemployment rates in Europe have been growing, although there has been wide heterogeneity between countries. At the same time, many European countries implemented labor market reforms, having taken a range of measures to deregulate labor markets and boost enterprise flexibility, lowering the quality of jobs [1]. Therefore, although reducing the unemployment rate was a priority, many workers became re-employed in poor-quality jobs. The growth of poor jobs related to economic crisis adds to its increase since the mid-1970s as a result of new forms of flexible employment. Consequently, more research is needed to contrast employment policies that often assume that the benefits of work are superior to any negative characteristics of work and the adverse effects of unemployment.

Our study provides innovative evidence regarding differences in mental wellbeing between unemployed people and people working in low and good quality jobs. Previous studies usually compared unemployed versus working people. Furthermore, we have analyzed the question from a perspective of gender inequalities, often lacking in studies that analyze work and health, despite the fact that positions of men and women in relation to the labor market are structurally different. Finally, we have explored the role of European welfare state typologies, adding the perspective of health to the long comparative research of welfare state and labor markets.

### Background

A large body of research has shown that unemployment has a detrimental effect on mental wellbeing [2,3]. Additionally, some studies have demonstrated the positive effect of gaining a job among unemployed people [4,5]. Nevertheless, previous studies analyzing job quality have indicated that low-quality jobs and precariousness are related to poor mental wellbeing [6]. 

Although there are a number of definitions, measures, and even indexes of job quality, a consensus is lacking on what constitutes job quality. For the purposes of this study, job quality is defined as the extent to which a job has work and employment-related factors that foster beneficial outcomes for the employee, particularly mental and physical well-being, as well as job satisfaction [7].

The few studies that question whether getting a job, regardless of its quality, improves the mental wellbeing of the unemployed population, tend to show that low-quality jobs have a similar or even worse effect than unemployment. These studies were conducted mainly outside Europe (Australia and the USA) [8,9,10,11,12]. The few studies that have been conducted in Europe have analyzed only a few specific countries [13,14]. However, differences in political, economic, and social contexts can determine differences in answer to this debate. As far as we know, no studies have analyzed this question at the European level, nor have they compared different welfare state typologies.

Despite the sexual division of labor, studies on gender inequalities and health in regards to job quality are scarce. A couple of recent studies have reported that, in countries with a traditional family model, gender differences in the impact of poor-quality jobs (mandatory long working hours and employment uncertainty) on mental wellbeing were related to the family context [15,16]. 

Additionally, many studies on unemployment and mental wellbeing have not examined potential gender differences. However, most studies have reported a stronger effect of unemployment on mental wellbeing among men [2], although one study reported a similar effect on both sexes [17] and another study reported a stronger association among women [18]. A recent study showing worse effects of unemployment on mental wellbeing among men from Ireland and a similar impact on men and women in Sweden highlighted the major influence of the context, including both the family and socioeconomic situations on the relationship between unemployment, gender, and mental wellbeing [19].

The extent to which the potential impact of unemployment and job quality have on mental wellbeing depends on the institutional context, i.e., the specific welfare regime. For instance, social protection during unemployment varies by welfare state regime, according to a complex mix of three principles: Universalism, social insurance, and means-testing. Bambra et al. [20] ranked 23 European countries grouped into five welfare state regimes, taking into account the generosity of benefits paid to the unemployed, the qualifying period and conditions, the duration of benefit payments, and the waiting period before activation of entitlement. The Scandinavian welfare regime was ranked first, and the Southern and Eastern regimes were ranked last. While the generosity of unemployment provision has been associated with a positive effect on the mental wellbeing of unemployed people [21,22], more restricted systems of benefits provision, such as means-tested benefits, have been associated with a stronger negative effect on mental wellbeing [23,24]. There is some evidence that active labor market programs (ALMP) have a positive impact on the mental wellbeing of unemployed people [25], and recent studies suggest that government spending on ALMP may counter the effect of recessions on suicide rates [26].

Despite the scarce evidence on job quality and welfare state regimes, the results indicate a similar direction. A recent review [27] suggested that, although globalization facilitates the growing precariousness of employment, more egalitarian welfare employment policies, such as those applied in Scandinavian countries, seem to act as a buffer against the negative effects of low-quality jobs on mental wellbeing.

Although seminal research on welfare state typology did not focus on the relationship between work and family policy, given the real-world interconnections between work and family obligations as well as gender and inequality, there is institutional compatibility of family and employment policies [28]. In fact, the classical classification of Esping-Andersen essentially overlaps with the family policy regimes of his three welfare states typologies [29], while Korpi proposes a similar typology in his analysis of welfare state policies related to gender and the family [30]. Recent studies taking differences in welfare state regimes into consideration suggest that the effect of unemployment on mental wellbeing is moderated by the labor market and family policies. In countries with a traditional family model and a labor market where female participation is not encouraged, while female engagement in caring for relatives and housework is promoted, most unemployed married women whose basic economic needs are guaranteed by their husband’s income could substitute the rewards formerly provided by their job for the rewards of their family-nurturing role. Conversely, among married men, the pressures related to their breadwinner role have a strong negative impact on their mental wellbeing [31]. In contrast, more egalitarian and generous welfare state regimes and dual breadwinner family models tend to show small or no gender differences in the impact of unemployment on mental wellbeing [19].

Similarly, the relationship between job quality and mental wellbeing is influenced by social institutions, particularly welfare programs and family structures. For instance, a sufficient and affordable supply of nursing homes and kindergartens might prevent difficulties in the work-life balance of workers with a given work schedule and dependent child. In contrast, workers with dependent relatives and in counties without supportive policies may be forced to find a low-quality job part-time, for example, often associated with poor working conditions [32]. Consequently, the interrelations between job quality and social institutions (welfare states and family structures) should always be explicitly considered when making international comparisons of job quality, especially when there are substantial differences in the social systems of the countries involved in the comparison [33].

Three points emerged in light of the revised evidence: (1) It is not clear whether, in Europe, working in a poor-quality job is better for mental wellbeing than being unemployed, (2) there is a lack of gender analysis that considers unemployment, job quality, and mental wellbeing jointly, which could show different gender patterns in this question, (3) the types of welfare state and the way in which labor and family models are configured can introduce differences into this question.

Therefore, the objectives of this study were: (1) To compare mental wellbeing between unemployed people and workers in different levels of job quality, and (2) to examine gender differences in mental wellbeing related to unemployment and job quality according to the welfare state typology in Europe. Our main hypothesis is that there are small or no differences in mental wellbeing between unemployed and employees in low-quality jobs, and that their mental wellbeing is worse than that of employees in good-quality jobs. We expect small differences in mental wellbeing between these groups in more generous welfare state regimes such as those in social-democratic countries. We also expect greater gender differences in the relationship between the employment situation and mental wellbeing in countries with a traditional family model and weak gender equality policies.

## 2. Materials and Methods

### 2.1. Data

This cross-sectional study was based on data from the European Social Survey (ESS) of 2010. The ESS is a representative sample of persons aged 15 years and older, who were resident in one of the 27 European countries. Every 2 years, the questionnaire included several questions about sociodemographic, health and wellbeing, education and occupation, social capital and social trust, and household circumstances. Additionally, the ESS-2010 included a specific module on work, family, and well-being. For the purpose of this study, a subsample of all employed and unemployed people were selected, currently salaried or unemployed, who were salaried workers in their last job, aged 16–64 years. To minimize possible reverse causation effects, unemployed individuals who left work for health reasons were excluded (*n* = 124), and those who had never been employed (*n* = 284). The final sample under analysis was composed of 8324 men and 7496 women.

### 2.2. Variables

#### 2.2.1. Mental Wellbeing

The dependent variable was measured through 3 items of the WHO-5 Well-Being Index (“I have felt cheerful and in good spirits”, “I have felt calm and relaxed” and “I have felt active and vigorous”) scored from 1 (all of the time) to 6 (at no time). Mental wellbeing was a measure of positive affect and is an important part of mental health that reflects the presence of positive feelings and positive functioning in life [34]. A score of >7 indicated poor mental wellbeing. The scale had good internal consistency (Cronbach’s alpha = 0.82). Although the original index contained 5 items, the 3-item scale had also been used in other studies [35].

#### 2.2.2. Employment Situation

People were grouped into 4 categories: (1) Unemployed, wanting a job but not actively looking for a job; (2) unemployed and actively looking for a job; (3) working in a low-quality job; and (4) working in a good-quality job. We included both groups of unemployed because non-active unemployed persons maintain their attachment to the labor market, and their lack of active search had been attributed to discouragement (they do not seek work actively because they believe there were no available jobs). At high unemployment rates, the unemployed may stop actively searching for work because they were discouraged by the high prevailing unemployment rate [36].

Job quality was measured with 5 indices according to the methodology of Green and Mostafa for the European Working Condition Survey (EWCS): Earnings, prospects, intrinsic job quality, working time quality, and participation and representation [7]. The specific questions selected from the survey to construct the indices are shown in Table S1.

A cluster analysis was carried out according to the 5 indices using K-means clustering. Two clusters emerged: Good-quality jobs and low-quality jobs. Table 1 shows the means and standard deviations of each of the 5 indexes according to the two groups of job quality (good and low) by sex, while Table 2 presents the median of the 5 indexes by sex and country group.

#### 2.2.3. Country Groups

Countries were grouped according to the classification used by Samuel and Hadjar in analyzing the ESS [37], based on the classical 3 categories of welfare state regimes defined by Esping-Andersen (Social-Democratic, Conservative, Liberal) [37], expanded by the Southern and the Post-socialist or Eastern welfare states. Countries were grouped in five categories: Conservative (Belgium, Germany, France, the Netherlands), Liberal (United Kingdom, Ireland), Eastern European (Bulgaria, Czech Republic, Estonia, Croatia, Hungary, Lithuania, Poland, Slovenia, Slovakia), Southern European (Cyprus, Spain, Greece, Portugal), and Social-Democratic (Denmark, Finland, Sweden).

#### 2.2.4. Other Variables Assessed in the Models

The models included 4 potential confounding variables: Negative affectivity, age (measured continuously), job category, and marital status. The personality trait characterized by sensitivity to negative stimuli (also termed neuroticism or negative affectivity) was known to correlate with both perceptions of work stress and with self-reported health [38]. In the same way, Coutts [25] recommended that in the analysis of the labor market status and health, adjustment should be made for the negative effect in any studies using self-reported health status indicators. This trait was assessed with 3 questions. Scores from 0 to 10 were added and categorized into 2 groups according to the median. Job category (a proxy of job qualification and socioeconomic position) [39] was assigned according to the respondent’s current occupation (or the last occupation among unemployed) and was determined based on the 2008 International Standard Classification of Occupations [40] 1-digit categories and subsequently grouped into 3 categories: Upper (1 and 2), medium (3–5), and lower (6–9). Marital status had 2 categories (married and unmarried).

### 2.3. Data Analysis

First, we tested for gender differences in the dependent and independent variables in each country typology at the bivariate level. The chi-square test for categorical variables and the t-test for age was used. Second, to examine the association between employment situation and mental wellbeing, multiple logistic regression models (reference category of the independent variable: Unemployed, wanting a job but not actively looking for a job) separated by sex and country group were fitted to calculate adjusted odds ratios (aOR) and 95% confidence intervals (95% CI). The models were adjusted for potential confounders (age, marital status, job category, and negative affectivity). All analyses included weights derived from the complex sample design.

## 3. Results

### 3.1. General Description of the Sample

A general description of the sample according to gender and country group is shown in Table 3. No gender differences in mental wellbeing were found in Eastern and Social-Democratic countries, where the highest and lowest levels of poor mental wellbeing, respectively, were found among both sexes (only women in Liberal countries scored higher in poor mental wellbeing than those in Eastern countries). In the remaining country typologies, the prevalence of poor mental wellbeing was higher among women, with the greatest gender differences being observed in Liberal countries. In both sexes, unemployment (both non-active and active) and low-quality jobs were more frequent in Southern and Eastern European countries. No gender differences in employment situations were found among Eastern and Social-Democratic countries.

### 3.2. Employment Situation and Mental Wellbeing

The results separated by sex and country groups are shown in Table 4. Among men, only in Conservative countries, low-quality jobs decreased the odds of having poor mental wellbeing compared with being unemployed (not active) (aOR = 0.46, 95% CI = 0.25–0.83). In the rest of the countries, there were no significant differences between non-active unemployment, active unemployment, and having a poor-quality job. In all countries, except for Social-Democratic countries, having a good-quality job reduced the likelihood of poor mental wellbeing among men compared with being unemployed (non-active), with aOR ranging from 0.19 (95% CI = 0.10–0.34) in Conservative countries to 0.43 (95% CI = 0.24–0.79) in Eastern European countries.

Among women, there were no significant differences between being unemployed-non-active, unemployed-active, and working in low-quality jobs in mental wellbeing in none of the countries. Women in Eastern and Southern European countries had a higher odds of improving their poor mental wellbeing when they worked in good-quality jobs compared with those who were unemployed (non-active) [in Eastern Europe aOR = 0.27 (95% CI = 0.14–0.51) and in Southern Europe aOR = 0.42 (95% CI = 0.22–0.84)].

## 4. Discussion

This study provides evidence that welfare state regimes and gender interact in the relationship between employment situation and mental wellbeing, taking job quality into account. As far as we know, this is the first study that used a large and representative sample of the EU-27. The study was conducted during the economic crisis and helped to clarify a key question: Is being employed always better for mental wellbeing than being unemployed?

This study has produced four main findings: (1) In both genders, there were no significant differences between unemployment (both non-active and active) and having a poor-quality job. The only exception was among men in Conservative countries where low-quality jobs were associated with better mental wellbeing than unemployment. (2) Only having a good-quality job reduced the likelihood of poor mental wellbeing compared with being unemployed (not active): This result was found among men in all countries (except Social-Democratic countries), that is, 19 out of 22 countries, and among women in Eastern and Southern European countries. Points 1 and 2 confirm our main hypothesis. (3) No association was found in either men or women in Social-Democratic countries. (4) Similar associations were found in both men and women in Southern and Eastern European countries, while strong gender differences were found in Conservative and Liberal countries, where men showed the strongest association, and no relationship was found in women. Points 3 and 4 are in line with our second and third hypotheses.

### 4.1. Employment Situation and Mental Wellbeing

Our results suggest that low-quality jobs and unemployment have a similar link to poor mental wellbeing and that having a job is insufficient for good mental wellbeing. Accordingly, this study highlights the importance of job quality in addition to merely promoting employment among the unemployed.

Our results are consistent with those found in Australia [8,12] and some European countries [10,13] but differ from those of two studies that showed opposite results in relation to low-quality jobs. The first study, conducted in the USA [11], reported that jobs with poor psychosocial conditions were associated with somewhat better mental wellbeing than unemployment. This discrepancy could be explained by differences in welfare state regimes and time periods. Although the USA is usually classified as a Liberal welfare regime, like the UK, unemployment benefits are lower [10]. In addition, the study was conducted in the second half of the 1990s, a period of relative economic prosperity. It has been reported that the stigma attached to unemployment (and related health penalty) is worse when population unemployment is low [41]. In the second study, a more recent and longitudinal study conducted in the UK, Chandola et al. [14] showed that mental wellbeing was worse in low-quality jobs compared with unemployment. These divergent results could be partially explained by the different designs and distinct variables used in our study. Chandola used chronic stress-related biomarkers as the dependent variable and included job anxiety (measured as the mean of six questions on job-related wellbeing) in the overall job quality variable.

Several theories can explain the mechanisms involved in the association of unemployment with poor mental wellbeing: The absence of latent functions provided by work (giving the day a time structure, providing opportunities for social contact with other people, contributing to status and personal identity for the individual, and providing an opportunity to strive toward collective purposes and shared experience) [42], economic deprivation [43], and uncertainty about the future employment situation [44]. Job quality was measured with an index that included social environment, earnings, and prospects, which are indicators related to the three models, respectively. In the conceptual framework of job quality proposed by Green and Mostafa [7] and used in our study, job quality is constituted by the features of jobs that meet workers’ needs from work. Therefore, poor-quality jobs could be associated with poor mental wellbeing in a similar way as unemployment, not providing for these human needs. One the other hand, while even a poor-quality job could provide a time structure for the day, the positive effect of this structure could be canceled out by some characteristics of this kind of job, such as working in the evenings, nights or weekends, or the obligation to work overtime at short notice. Finally, a lack of support and help from coworkers in low-quality jobs could be similar to the social isolation associated with unemployment.

### 4.2. Welfare State Regimes

In Social-Democratic countries, there was no association between the employment situation and mental wellbeing. This result could be explained by high unemployment protection and higher expenditure on employment policies. Financial security provided by generous unemployment benefits would lead to better mental wellbeing, while scarcity was related to poverty [22]. If unemployed people have their minimum financial needs covered, they could benefit from positive health-related behaviors, such as having extra time for family, social relationships, and exercise, as well as lower work stress and exposure to other occupational hazards [45]. Moreover, it has been argued that ALMPs can improve the mental wellbeing of the unemployed, at least to some extent, matching the psychosocial functions of employment itself [46,47]. Both policies, generous unemployment protection, and investment in ALMP have been related to increased mental wellbeing not only among the unemployed but also among working people [48], in the latter case, probably because they feel less threatened if they lose their jobs.

In addition, generous social protection schemes would help people in poor-quality jobs cope with stressful and adverse working conditions. For instance, the presence and extent of dismissal-protection laws could reduce job insecurity. In addition, Social-Democratic countries have stronger and more comprehensive workplace regulations than other European countries [49]. This is congruent with the best score of the five indexes of job quality in these countries (Table 3).

In both sexes, unemployed people and workers in low-quality jobs were more likely to report poor mental wellbeing in Southern European countries, whose welfare state regimes are characterized by a highly fragmented social protection system with gaps in unemployment protection and regulation of employment security [50]. In addition to financial insecurity and the risk of poverty associated with unemployment in these less generous welfare state regimes, high unemployment rates during the recent economic crisis in those countries reduced the employability of people with health problems, resulting in their accumulation among the unemployed persons.

In addition, previous research has shown a strong association between poor psychosocial working conditions and mental wellbeing among Southern European countries that have been related to less favorable labor regulations, for instance, in relation to employment security [48]. Moreover, successive labor reforms, implemented in many of these countries as a consequence of the economic crisis, have further reduced job quality [1]. Low scores in some indexes of job quality in these countries point in this direction (intrinsic job quality, prospects, participation, and representation) (Table 3). In relation to participation and representation, high unemployment rates have been related to increasing precariousness, limiting workers’ bargaining power both collectively and individually. During a crisis, unions cannot counter or are pushed to accept labor market reforms that tend to increase employment precariousness. At the individual level, many labor market survivors will feel insecure about their own jobs and will accept a decline in employment and working conditions to remain employed [6].

Notable findings were the high percentage of active unemployed in Southern countries (20.9% in men and 16.6% in women) and the lack of differences in mental wellbeing between them and non-active unemployed.

Similar results were found in Eastern European countries. In comparison with the other member states of the European Union, the social protection during unemployment in Eastern European regimes was less generous (in terms of benefits paid to the unemployed, the duration of benefit payments, and the waiting period before entitlement is activated) [20]. Another factor was the rather high unemployment levels in those countries (in 2010, four of the six countries in the EU-28 with the highest unemployment rates in the Eastern group: Slovakia, Estonia, Lithuania, and Latvia, with values higher than 14%) [51]. Therefore, as previously mentioned in relation to Southern European countries, the poor mental wellbeing among unemployed could be explained by the low social unemployment benefits, and high unemployment rates.

In addition, it is possible that working and employment conditions were worse among low-quality jobs in Eastern European countries, as suggested by the fact that most of the index values of job quality were lower in these countries (Table 3). For example, the minimum wage of all the new member states of the EEC (except Slovenia) fell behind those of developed capitalist democracies. Moreover, one of the features of the Eastern European welfare regime is the low trade union density and representation of employees [52]. Of importance, the highest percentage of poor-quality jobs was observed in Eastern European countries; indeed, in those countries, low-quality jobs were the most frequent employment situation (44.4% of men and 43.3% of women). Similarly, using the fourth EWCS, Puig-Barrachina et al. reported high levels of employment precariousness among Eastern and Southern European countries [53].

The results in the Conservative and Liberal welfare regimes showed strong gender differences, which are discussed in the next section.

### 4.3. Gender

Significant gender differences were identified among Conservative and Liberal countries. Men from these countries showed the strongest relationship between good-quality jobs and mental wellbeing compared with unemployed men, while no association was found between employment situation and mental wellbeing among women.

The highest prevalence of poor mental wellbeing was found among unemployed men from Conservatives countries, mainly non-active unemployed, but also active unemployed (together with men from Eastern countries). These results were similar to those reported by Bambra [20], who suggested a couple of reasons that could explain, at least partially, our results. On the one hand, although these countries ranked high in the generosity of unemployment benefits, these benefits were often earnings-related and aiming to maintain existing social patterns, thus restricting the benefits of high-job categories. On the other hand, the breadwinner role in the traditional family model could contribute to a stigmatization of unemployed men. The high prevalence of poor mental wellbeing among non-active unemployed men (50.0%) seems to point in this direction. Although we cannot rule out an alternative explanation relating poor health to a non-active search for any job, this potential bias is likely to be minimized by not including people in unemployment for health reasons.

In contrast, men from Conservative countries constituted the only group in which mental wellbeing was better in low-quality jobs among the unemployed. These countries were characterized by a deep dualization of the workforce, where outsiders (people with a high risk of unemployment and atypical and precarious employment) [54] were at specific disadvantages, such as poor job prospects, poverty, welfare losses, and a lack of social and political integration [55]. Insiders, on the other hand, were those in stable and standard employment, which would explain their better mental wellbeing. In this regard, we expected a similar level of poor mental wellbeing among unemployed men and men in low-quality jobs, but the results showed that the latter were in better health than unemployed. This result seems to reinforce the poor situation of non-active unemployed men, who could benefit from having a job, even of poor quality.

Contrary to men, non-active unemployed women in Conservative countries showed low levels of poor mental wellbeing. In countries with low levels of support for female participation in the labor force and with a strong traditional family model, non-active unemployment could be less damaging to women’s mental wellbeing, especially those with family responsibilities, who can devote more time to their role as caregivers [31]. A question emerges in relation to non-binary gender approaches in countries with a strong male breadwinner model, among people who do not conform to these prescribed roles. For example, women who decide to become financially independent, without a male partner, and with or without children; or women who decide to live with other women. As the welfare state is designed according to traditional gender roles, what is the impact of the employment situation on their mental wellbeing? This is a question that deserves future studies. In this regard, surveys should include measures of gender on a spectrum [56].

The Liberal welfare regime is characterized by comparably low levels of welfare provision, means testing, and a deregulated labor market. The results among unemployed men could be related to less generous unemployment benefits and a deeply traditional family model. In addition, the polarization of jobs in Liberal regimes that was reported in many studies [57], and that seemed to be strongest among males [16], could explain the different results between men employed in low and high-quality jobs. In this regard, it should be noted that there were no differences between active unemployed men and men in low-quality jobs.

Non-active unemployed women from Liberal and Eastern regimes showed poorest mental wellbeing along with men from Conservative regimes. If stigmatization related to the breadwinner role among men could be related to these results among men, as previously mentioned, in the case of women, a possible explanation could be the low social insurance benefits characterizing those regimes that penalize women as they have lower salaries than men (for example, the net replacement rate in the United Kingdom was 54% and in Hungary 49% [20]). In addition, Eastern European countries have a tradition of long weekly hours, and overtime payments often constitute a regular and substantial element of wage packages [58]. In these countries, labor markets attach a high value to those employees who are generally willing to work long hours. However, workplaces typically remain inflexible with workers needing to work fewer hours, thus putting employees with caring responsibilities, who are typically women, at a disadvantage. There is a ‘penalty’ for motherhood in the labor market that is increased through the institution of a long period of parental leave, which is typically taken by mothers. As a result of refamiliarization, precarious work has acquired a specific form for women who accept precarious jobs when they have caring responsibilities as a temporary strategy that may turn into a trap excluding them from a better job [59,60].

Previous research has shown a stronger relationship between unemployment and poor mental wellbeing among men in some Southern European countries than among women, related to men’s breadwinner role in traditional family models [31]. Consequently, we would expect a lower association between good-quality jobs and good mental wellbeing among women, but the results were similar in both genders. It has been reported that during the last economic crisis, European women (especially those in South European countries), unlike men, have increased their labor market participation by entering the labor market or increasing their working hours in order to offset the drop in earnings of their male partners. Their economic needs and vulnerability reduce their bargaining power for better employment conditions. Additionally, these results can be explained by the known greater domestic and homecare workload among women in those countries in a context of minimal public support for the care of dependents and men’s limited contribution to housework [61]. Of note, there were no differences in mental wellbeing between active unemployed women and women in poor-quality jobs. This result reinforces the argument that poor employment and working conditions can be as harmful to mental wellbeing as unemployment, mainly in countries with weak public support policies, both labor and family.

### 4.4. Strengths and Limitations

Our results could be limited by the cross-sectional design and the data. The possibility of reverse causation must be considered, but in the case of unemployed people, this question was dealt with by excluding those who were unemployed for health reasons, as well as those never employed. This restriction could be more important in non-active unemployed persons and could have contributed to select discouraged unemployed into this group. This situation is probably more frequent in times of crisis. In addition, reverse causation can explain, at least in part, the association between mental wellbeing and low job quality. However, longitudinal studies indicate that the practical importance of this effect might be limited in the case of unemployment [2] and low-quality jobs [14].

The measurement of job quality is controversial [33]. We adapted the ESS data to the methodology proposed by Green and Mostafa for the EWCS, which has a conceptual framework and a solid theoretical development. A limitation of our study is the lack of data on the physical environment. Although this is an important characteristic of job quality, its influence on mental wellbeing is probably lower than that of the three other dimensions of the intrinsic job quality index (skills and discretion, social environment, work intensity). Additionally, the relative contribution of the total quality value of the physical environment is less high (it is one of the four dimensions of intrinsic job quality index, which in turn is one of the five quality indexes used).

A further limitation is that the measures of mental wellbeing and job quality were self-reported. As such, response-bias or endogeneity may play a role in the significant associations found between them. This possibility was minimized in the analyses by including negative affect as a potential covariate.

Despite these limitations, this study has a number of strengths. We used a large, representative sample from Europe that allowed us to compare different welfare state typologies and desegregate the analysis by gender. The ESS is one of the few surveys to compare health and mental wellbeing between unemployed and employed persons, with sufficient information to permit in-depth measurement of job quality. Working condition surveys, such as the EWCS, are highly effective in measuring job quality but do not include unemployed persons. Workforce surveys have a similar limitation, provide less information on job quality, and do not usually include health and mental wellbeing. Finally, health surveys include unemployed and health and mental wellbeing, but usually, have few questions on job quality. A final strength is that job quality was measured using five dimensions of job quality, unlike most studies that measure only job satisfaction.

## 5. Conclusions

Our study questions the widespread belief that any employment, even in poor-quality jobs, is associated with better mental wellbeing compared with being unemployed. The study highlights the need to take job quality into account, in addition to creating jobs in contexts of economic crisis. Strong gender and welfare state differences were identified: No association was found between employment situation and mental wellbeing in Social-Democratic countries in either men or women; unemployed and people in low-quality jobs in Southern and Eastern European countries—both men and women—showed similar levels of poor mental wellbeing; and strong gender differences were found in Conservative and Liberal countries, where men showed the strongest association between employment situation and mental wellbeing, while no relationship was found among women. The main mechanisms to explain these findings could be social protection during unemployment, labor market and workplace regulations, and family models. Additionally, the crisis and policies aimed at mitigating its effects have added other elements to the complex interaction between work and employment conditions, family models, and gender inequalities.

## Figures and Tables

**Table 1:** Five indexes of two groups of job quality (mean and standard deviation) by sex. People currently employed. European Social Survey, 2010/11.

Indexes	Men		Women	
	Good Quality	Low Quality	Good Quality	Low Quality
	*n* = 3793	*n* = 2300	*n* = 3256	*n* = 2177
Earnings	60.4 (25.5)	43.1 (27.6) **	57.2 (26.8)	38.0 (27.0) **
Prospects	73.6 (14.9)	52.4 (17.7) **	71.8 (15.6)	50.8 (17.7) **
Intrinsic job quality	61.0 (10.6)	42.4 (12.1) **	61.0 (10.5)	41.7 (12.8) **
Working time quality	52.8 (17.4)	42.1 (17.7) **	59.0 (17.0)	50.9 (17.8) **
Participation and representation	41.2 (22.3)	20.4 (18.1) **	41.1 (22.4)	20.6 (18.2) **

** *p* < 0.01. *p*-values compare indexes in good- and low-quality job groups. The mean and standard deviation refers to the score from 0 to 100 for each of the five indices.

**Table 2:** Five indexes of job quality (medians) by sex and country group. People currently employed. European Social Survey, 2010/11.

Indexes	Conservative		Liberal		Eastern		Southern		Social-Democratic	
	Men	Women	Men	Women	Men	Women	Men	Women	Men	Women
	*n* = 3019	*n* = 2722	*n* = 1042	*n* = 938	*n* = 1138	*n* = 1038	*n* = 848	*n* = 765	*n* = 340	*n* = 351
Earnings	51.6	42.4 ***	55.3	54.9	32.9	28.2 *	44.9	42.7	58.5	47.0
Prospects	80.6	77.4 ***	81.1	78.0	72.4	70.8	77.5	73.4 **	86.4	87.2
Intrinsic job quality	69.2	64.9 ***	62.6	62.7	53.4	53.0	51.6	44.8	77.2	79.9
Working time quality	50.7	66.1 ***	41.0	65.0 ***	42.1	60.2 ***	49.5	59.1 ***	59.5	67.0 **
Participation and representation	16.5	13.9 ***	13.0	16.4 ***	12.4	14.0 **	12.9	11.1	29.0	34.7 *

* *p* < 0.05. ** *p* < 0.01. *** *p* < 0.001. *p*-values compare gender differences in indexes according to the Mann–Whitney U test. The median refers to the score from 0 to 100 for each of the five indices.

**Table 3:** General description of the population (in percentages) by sex and country group. European Social Survey, 2010/11.

Title	Conservative		Liberal		Eastern European		Southern European		Social-Democratic	
	Men	Women	Men	Women	Men	Women	Men	Women	Men	Women
	*n* = 3543	*n* = 3236	*n* = 1343	*n* = 1198	*n* = 1690	*n* = 1493	*n* = 1354	*n* = 1174	*n* = 394	*n* = 395
Poor mental wellbeing	20.2	22.6 *	17.1	28.3 ***	24.1	26.2	14.5	18.2 *	13.4	17.8
Employment situation										
Unemployed-non-active	1.5	2.0 ***	3.3	2.7 ***	4.9	4.2	4.6	6.0*	2.6	1.5
Unemployed-active	6.4	6.4	10.7	6.3	12.0	10.0	20.9	16.6	6.8	4.8
Low-quality job	29.2	35.7	33.3	31.6	44.4	43.3	36.4	38.6	16.5	16.0
Good-quality job	62.9	55.9	52.7	59.4	38.7	42.6	38.1	38.8	74.0	77.7
Married	56.9	54.5	52.9	47.5 **	61.4	59.1	56.4	55.8	47.6	48.0
Job category										
Upper	23.3	19.7 ***	33.5	24.4 ***	22.4	28.2 ***	14.6	17.7 ***	27.8	26.2 ***
Medium	35.3	64.4	24.0	61.1	22.0	48.0	34.1	58.6	30.9	63.9
Lower	41.3	15.9	42.5	14.5	55.6	23.8	51.2	23.7	41.3	9.9
Negative affectivity	21.5	20.2	14.3	15.2	38.3	36.0	26.6	27.1	6.7	4.1
Age (SD)	41.4 (11.5)	42.1 (11.5) *	38.7 (12.3)	40.2 (11.6) **	40.0 (11.9)	40.7 (11.1)	39.7 (10.6)	39.9 (10.1)	41.7 (11.9)	43.0 (11.7)

* *p* < 0.05. ** *p* < 0.01. *** *p* < 0.001. *p*-values compare men and women in each country group.

**Table 4:** Association of employment situation with poor mental wellbeing by sex and country group. European Social Survey, 2010/11.

**Men**										
	**Conservative** *n* = 3543		**Liberal** *n* = 1343		**Eastern European** *n* = 1690		**Southern European** *n* = 1354		**Social-Democratic** *n* = 394	
	%	aOR (95% CI)	%	aOR (95% CI)	%	aOR (95% CI)	%	aOR (95% CI)	%	aOR (95% CI)
Unemployed-non-active	50.0	1	38.5	1	34.3	1	21.2	1	20.0	1
Unemployed-active	35.9	0.60 (0.32–1.15)	21.5	0.51 (0.23–1.12)	35.8	1.07 (0.57–2.01)	27.8	1.57 (0.75–3.25)	19.2	0.82 (0.13–5.22)
Low-quality job	29.6	0.46 (0.25–0.83) *	23.6	0.53 (0.26–1.10)	26.6	0.73 (0.41–1.29)	14.6	0.68 (0.33–1.43)	29.0	1.26 (0.24–6.59)
Good-quality job	13.4	0.19 (0.10–0.34) ***	10.2	0.20 (0.09–0.42) ***	17.6	0.43 (0.24–0.79) **	7.2	0.33 (0.15–0.73) **	8.6	0.33 (0.07–1.67)
**Women**										
	**Conservative** *n* = 3236		**Liberal** *n* = 1198		**Eastern European** *n* = 1493		**Southern European** *n* = 1174		**Social-Democratic** *n* = 395	
	%	aOR (95% CI)	%	aOR (95% CI)	%	aOR (95% CI)	%	aOR (95% CI)	%	aOR (95% CI)
Unemployed-non-active	28.8	1	45.5	1	46.0	1	25.4	1	33.3	1
Unemployed-active	33.3	1.43 (0.74–2.75)	53.8	1.58 (0.59–4.27)	41.7	0.97 (0.49–1.92)	22.0	0.85 (0.42–1.72)	33.3	0.79 (0.07–9.69)
Low-quality job	29.4	1.18 (0.66–2.13)	34.7	0.79 (0.32–1.92)	31.7	0.60 (0.33–1.09)	22.0	0.80 (0.42–1.54)	32.2	0.64 (0.06–6.59)
Good-quality job	16.9	0.70 (0.38–1.26)	23.5	0.49 (0.20–1.19)	16.9	0.27 (0.14–0.51) ***	13.4	0.42 (0.22–0.84) *	13.1	0.19 (0.02–1.88)

* *p* < 0.05. ** *p* < 0.01. *** *p* < 0.001. Notes: % refers to the prevalence of poor mental wellbeing in each category. aOR: All models are adjusted by marital status, job category, negative affectivity, and age.

## Supplementary Materials

The following are available online at https://www.mdpi.com/1660-4601/16/23/4799/s1, Table S1: Composition of the five indices of job quality. Items of the European Social Survey, 2010/11.
