# Supplementary material for: Is Being Employed Always Better for Mental Wellbeing Than Being Unemployed? Exploring the Role of Gender and Welfare State Regimes during the Economic Crisis
Source: Int J Environ Res Public Health. 2019 Nov 29;16(23):4799. doi: 10.3390/ijerph16234799 (PMC6926996; doi:10.3390/ijerph16234799)
Supplement: Supplementary file 1 [file ijerph-16-04799-s001.pdf]

International Journal of
Environmental Research and Public Health

MDPI

Table S1. Composition of the five indices of job quality. Items of the European Social Survey, 2010/11.

1. EARNINGS

FAIRNESS OF WAGES

- Considering all my efforts and achievements in my job, I feel I get paid appropriately.

2. PROSPECTS

JOB SECURITY

- My job is secure.

CAREER PROGRESSION

- My opportunities for advancement are good.

CONTRACT QUALITY

- Do you have a work contract of unlimited duration, limited duration or, do you have no contract?

3. INTRINSIC JOB QUALITY

SKILLS AND DISCRETION

- During the last twelve months, have you taken any course or attended any lecture or conference to improve your knowledge or skills for work?
- How much of this training or education was paid for by your employer or firm?
- There is a lot of variety in my work.
- My job requires that I keep learning new things.
- How much the management at your work allows/allowed you to decide how your own daily work is/was organized?
- How much the management at your work allows/allowed you to influence policy decisions about the activities of the organization?
- How much the management at your work allows/allowed you to choose or change your pace of work?

Education level required in the occupation

- If someone was applying nowadays for the job you do now, would they need any education or vocational schooling beyond compulsory education?
- About how many years of education or vocational schooling beyond compulsory education would they need?

Experience needed to do the job

- If somebody with the right education and qualifications replaced you in your job, how long would it take for them to learn to do the job reasonably well?

GOOD SOCIAL ENVIRONMENT

- I can get support and help from my co-workers when needed

WORK INTENSITY

- My job requires that I work very hard.
- I never seem to have enough time to get everything done in my job.

**4. WORKING TIME QUALITY**
DURATION
• Regardless of your basic or contracted hours, how many hours do/did you normally work a week (in your main job), including any paid or unpaid overtime?
CONDUCTIVE SCHEDULLING
• How often does your work involves working at evenings or nights?<br>• How often does your work involves working at weekends?
DISCRETION OVER WORKING TIME ARRANGEMENTS
• I can decide the time I start and finish work.
SHORT-TERM FLEXIBILITY
• How often does your work involves having to work overtime at short notice?
**5. PARTICIPATION AND REPRESENTATION**
• At your workplace are there regular meetings between representatives of the employer and employees, in which working conditions and practices can be discussed?<br>• How much influence would you say these discussions generally have on decisions that affect your working conditions and practices?<br>• How much influence would you say that trade unions at your workplace generally have over decisions that affect your working conditions and practices?
